# Development and Validation of a Routine Electronic Health Record-Based Delirium Prediction Model for Surgical Patients Without Dementia: Retrospective Case-Control Study

**DOI:** 10.2196/59422

**Published:** 2025-01-09

**Authors:** Emma Holler, Christina Ludema, Zina Ben Miled, Molly Rosenberg, Corey Kalbaugh, Malaz Boustani, Sanjay Mohanty

**Affiliations:** 1 Department of Surgery Indiana University School of Medicine Indianapolis, IN United States; 2 Department of Epidemiology & Biostatistics Indiana University Bloomington Bloomington United States; 3 Department of Electrical & Computer Engineering Lamar University Beaumont, TX United States; 4 Department of Medicine Indiana University School of Medicine Indianapolis, IN United States

**Keywords:** delirium, machine learning, prediction, postoperative, algorithm, electronic health records, surgery, risk prediction

## Abstract

**Background:**

Postoperative delirium (POD) is a common complication after major surgery and is associated with poor outcomes in older adults. Early identification of patients at high risk of POD can enable targeted prevention efforts. However, existing POD prediction models require inpatient data collected during the hospital stay, which delays predictions and limits scalability.

**Objective:**

This study aimed to develop and externally validate a machine learning-based prediction model for POD using routine electronic health record (EHR) data.

**Methods:**

We identified all surgical encounters from 2014 to 2021 for patients aged 50 years and older who underwent an operation requiring general anesthesia, with a length of stay of at least 1 day at 3 Indiana hospitals. Patients with preexisting dementia or mild cognitive impairment were excluded. POD was identified using Confusion Assessment Method records and delirium International Classification of Diseases (ICD) codes. Controls without delirium or nurse-documented confusion were matched to cases by age, sex, race, and year of admission. We trained logistic regression, random forest, extreme gradient boosting (XGB), and neural network models to predict POD using 143 features derived from routine EHR data available at the time of hospital admission. Separate models were developed for each hospital using surveillance periods of 3 months, 6 months, and 1 year before admission. Model performance was evaluated using the area under the receiver operating characteristic curve (AUROC). Each model was internally validated using holdout data and externally validated using data from the other 2 hospitals. Calibration was assessed using calibration curves.

**Results:**

The study cohort included 7167 delirium cases and 7167 matched controls. XGB outperformed all other classifiers. AUROCs were highest for XGB models trained on 12 months of preadmission data. The best-performing XGB model achieved a mean AUROC of 0.79 (SD 0.01) on the holdout set, which decreased to 0.69-0.74 (SD 0.02) when externally validated on data from other hospitals.

**Conclusions:**

Our routine EHR-based POD prediction models demonstrated good predictive ability using a limited set of preadmission and surgical variables, though their generalizability was limited. The proposed models could be used as a scalable, automated screening tool to identify patients at high risk of POD at the time of hospital admission.

## Introduction

Postoperative delirium (POD) is a common and serious surgical complication that affects 15%-50% of older surgical patients [1-3]. POD is characterized by acute fluctuations in consciousness and has a complex etiology thought to be caused by interactions between predisposing (eg, individual vulnerability) and precipitating (eg, acute illness or surgery) factors [4]. Common predisposing factors include older age, preexisting cognitive impairment, poor physical functioning, alcohol abuse, smoking, and depression [5-8]. Risk factors unique to surgical settings include the type of surgery (eg, major vascular procedures), emergent status, case complexity, and perioperative medications [6,7,9,10]. Despite being an acute condition, delirium is associated with long-term cognitive and physical impairment, institutionalization, and death [4,11]. However, up to 40% of cases may be preventable, and multicomponent, nonpharmacologic interventions may be effective in reducing incidence and health care costs [12,13].

Early and accurate POD risk prediction can inform prevention and enable targeted intervention and resource planning efforts. Fortunately, the widespread availability of electronic health record (EHR) data and advancements in machine learning offer an opportunity to develop accurate, low-cost, and scalable screening tools for POD risk. Several machine learning-based POD prediction models have been developed, reporting areas under the curve (AUROCs) ranging from 0.71 to 0.86 [14-26]. However, the models with the highest AUROCs have important limitations that hinder their practical application. First, they focus on specific patient subsets (ie, intensive care unit (ICU) patients, cardiac surgery), which restricts their generalizability to general surgical populations. Second, population-specific models necessitate separate models for each subpopulation, making implementation cumbersome and resource intensive. Finally, many of these models require inpatient data that take hours or days to accumulate, delaying risk assessment and potential interventions. A small number of studies have developed POD prediction models for general surgical populations; however, these models still incorporate nonroutine clinical data (eg, inpatient nursing assessments) that require time to collect and may not be universally available [14-18,27].

These limitations highlight the need for a model that can predict POD in a diverse surgical population using readily available preoperative data, as it could provide an early, inexpensive, and scalable prescreening tool to identify patients who may benefit from additional monitoring or preventative measures. In this study, we developed and externally validated a machine learning model that can accurately predict POD in surgical patients at the time of hospital admission using only routine EHR data. We also identified preoperative EHR-based predictors of POD and determined how preoperative surveillance length affected model performance.

## Methods

### Ethical Considerations

This study was approved by the Indiana University (IU) Institutional Review Board (#15767) and adhered to the reporting standards described in the Transparent Reporting of Individual Prognosis or Diagnosis (TRIPOD) guidelines [27].

### Study Data and Cohort Selection

Diagnoses, medication orders, surgery, and other inpatient clinical records (eg, nursing assessments) were extracted from the IU Health electronic data warehouse. IU Health, a nonprofit health system with the largest physician network in the state of Indiana, includes 17 hospitals and dozens of outpatient facilities and performs approximately 115,000 surgeries per year [28]. We identified all surgical hospitalizations for patients aged 50 years and older who underwent surgery requiring general anesthesia at an IU Health facility between January 1, 2014, and December 31, 2021; had a length of stay of at least 1 day; and did not have preexisting dementia. Hospitalizations of patients with preexisting dementia (defined as having a dementia diagnosis code or an order for an antidementia medication before admission; see Table S1 in Multimedia Appendix 1) were excluded because dementia is known to be the single-strongest predictor of delirium [6]; models are not needed to forecast risk. For a hospitalization to be eligible, the patient had to have at least 1 IU Health encounter (defined as any interaction with an IU Health facility, eg, outpatient, inpatient, or emergency department visits) in the year before admission and have at least 1 diagnosis or medication record during that period. If no sex, race, or age data were available across all of a given patient’s hospitalizations, that patient was excluded.

This study followed a retrospective case-control design where nondelirium (ie, control) hospitalizations were matched to delirium (ie, case) hospitalizations by sex, race, age within 3 years, and admission year within 3 years. We matched on these variables to ensure the age distribution for cases and controls was equalized across race and sex groups. As a result, age was less important to the model, and biases within strata of race and sex were minimized. Because matching was done at the hospitalization level rather than the patient-level, it was possible for case and control hospitalizations belonging to the same patient to be matched.

Hospitalizations where the patient developed POD were designated as cases. POD was defined as at least 1 positive Confusion Assessment Method (CAM) [29] nursing assessment or a delirium *International Classification of Diseases, Ninth Revision* (ICD-9)/*International Classification of Diseases, Tenth Revision, Clinical Modification* (ICD-10-CM) code (see Table S2 in Multimedia Appendix 1) recorded during the hospital stay. The CAM is a validated diagnostic algorithm with an overall sensitivity of 94% and a specificity of 89% [30]. Hospitalizations where delirium was present at the time of admission were excluded because the model is intended to predict POD. Hospitalizations without delirium or any nurse-documented confusion (ie, cognitive assessments reporting that the patient was disoriented, confused, or did not follow commands) were eligible to be selected as controls. Visits that did not have documented delirium (ie, delirium ICD code or positive CAM) but did have nurse-documented confusion were excluded from the control pool to ensure controls were not actually misclassified cases; confusion (without delirium) could possibly represent subsyndromal delirium. If a case had more than 1 potential control, a control was randomly selected. For each eligible visit, the index date was defined as the date of hospital admission. We used the following set of sociodemographic, surgery, diagnosis, and medication variables to build our predictive models.

#### Variables

Sociodemographic variables included age, patient-reported sex, and patient-reported race (categorized as Black, White, Asian, other, or unknown for analytic purposes), and insurance type. The insurance type was ascertained during each index visit and categorized as commercial, government (Medicare or Medicaid), self-pay, or other/unknown. Smoking status at the time of surgery was extracted from the EHR and categorized as “current,” “former,” or “never smoker.” The BMI was obtained from the visit nearest to the index. The initial American Society of Anesthesiologists (ASA) class and emergency surgery status (defined as operations with an ASA class of 5 or E) were also included. Surgical specialty was assigned based on National Surgical Quality Improvement Program inclusion and exclusion criteria [31]**.** If a patient underwent 4 or more procedures falling under 2 or more distinct specialties, the visit was categorized as “multispecialty.”

Diagnosis variables were generated using ICD-9/ICD-10-CM codes. Binary variables were created for each of the 31 Elixhauser disease groups using Quan et al [32] coding scheme and Elixhauser mortality scores were calculated for each patient using van Walravan weights [32-34]. We also created binary variables for other diagnoses potentially associated with increased risk of delirium, including previous delirium, cerebrovascular disease (CVD), previous traumatic brain injury (TBI), and sensory impairment (Table S3 in Multimedia Appendix 1). We derived a composite variable representing the total comorbidity burden by calculating the sum of the number of unique ICD codes (at the 3-digit level) a patient had prior to each index date. Variables for the number of ICD codes belonging to the ICD-10 group Z00-Z99 (factors influencing health status and contact with health services) and their ICD-9 equivalents were also included based on prior literature [14], grouped as follows: Z00-Z13, Z16, Z17, Z18, Z20-29, Z30-39, Z40-53, Z55-65, Z69-76, and Z77-99.

Medication variables were generated using medication order data. Anticholinergic (ACh) medications were identified using the Anticholinergic Cognitive Burden (ACB) scale, a well-established tool that categorizes medications based on the strength of their ACh activity [35]. Three ACh medication variables were developed representing the total number of orders for drugs with an ACB score of 1, 2, and 3, respectively. We also included other non-ACh medication variables as predictors. Since medication orders were retrieved from multiple health care institutions, a unified mapping of medication names to a drug taxonomy was not available. Instead, we mapped each medication in the medication orders to the Anatomical Therapeutic Chemical (ATC) classification codes [36]. The ATC drug classification system is hierarchical with multiple sublevels and maintained by the World Health Organization. For this study, all 14 main groups (eg, A: alimentary tract and metabolism; B: blood and blood-forming organs; C: cardiovascular system) and the first-level subgroup were included (eg, A01: stomatological preparations; A02: drugs for acid-related disorders). For each patient, the count of medication orders (excluding AChs, which were derived separately, as described before) associated with a given ATC subgroup was calculated over the preindex assessment period. We also created a variable summing the total number of medication orders before each admission to capture polypharmacy.

#### Model Development and Evaluation

Three IU Health institutions were selected for this study. Institutions A, B, and C had the first-, second-, and third-greatest number of delirium cases, respectively. Institution-specific models were developed using data derived from the following preindex surveillance periods: 3 months before admission, 6 months before admission, and 1 year before admission. The purpose of training these separate models was to provide an understanding of how the training data and surveillance period impact the models’ ability to predict POD and generalizability. Prior to training, each model’s data were split into training (80%) and holdout (20%) sets, while maintaining a 1:1 ratio of cases and controls to avoid class imbalance. Imbalanced data are problematic in classification tasks because the model will focus on learning the characteristics of the majority class. As a result, the model may achieve high accuracy but fail to accurately identify the minority class.

In this study, 6 demographic variables, 4 surgical variables, 49 diagnosis variables, and 84 medication variables were included for a total of 143 features. Categorical variables were one-hot encoded (ie, converted into dummy variables), and continuous variables were standardized such that they each had a mean of 0 and an SD of 1. We initially explored several different machine learning models to predict whether patients would develop POD after surgery. In addition to traditional logistic regression, a parametric model, we also tried random forest, extreme gradient boosting (XGB), and a multilayer neural network because they can learn complex nonlinear relationships between variables. Optimal hyperparameters for each model were selected using a grid search with 5-fold cross-validation. Each candidate model was evaluated by calculating the area under the receiver operating characteristic curve (AUROC) on its holdout set using data from 1 year before hospital admission, and the model with the highest AUROC was selected as the final model. XGB outperformed the other candidate classifiers in all cases.

After model selection, XGB models trained on data from institution A (referred to as XGB_A_) were internally validated on holdout data from institution A and externally validated using holdout data from institutions B and C. Similarly, models trained on data from institutions B and C (referred to as XGB_B_ and XGB_C_, respectively) were internally validated on holdout data from institutions B and C and externally validated using data from institutions A and C and A and B, respectively. The predictive performance of each model was evaluated on the holdout and external validation data by creating 1000 bootstrapped samples without replacement, calculating the AUROC, sensitivity, specificity, positive predictive value (PPV), and negative predictive value (NPV) in each sample and then averaging them across all samples. We also generated predictions for nondelirium visits with nurse-documented confusion (which were excluded from training) to examine how the models handle patients with possible subsyndromal delirium. The default threshold of 0.50 was used for predictions. Shapley Additive Explanation (SHAP) [37] was used to determine the most important features, and model calibration was assessed using calibration curves. All analyses were completed using R version 4.3.2 (R Foundation for Statistical Computing).

## Results

### Study Cohort

**Figure 1:**
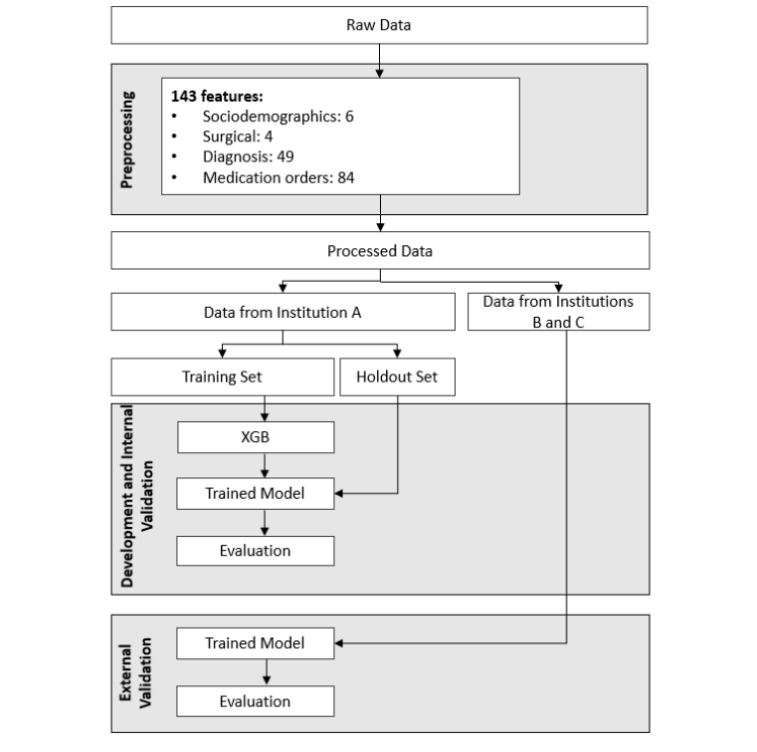
Workflow for the development and validation of the model using data from institution A. XGB: extreme gradient boosting.

**Figure 2:**
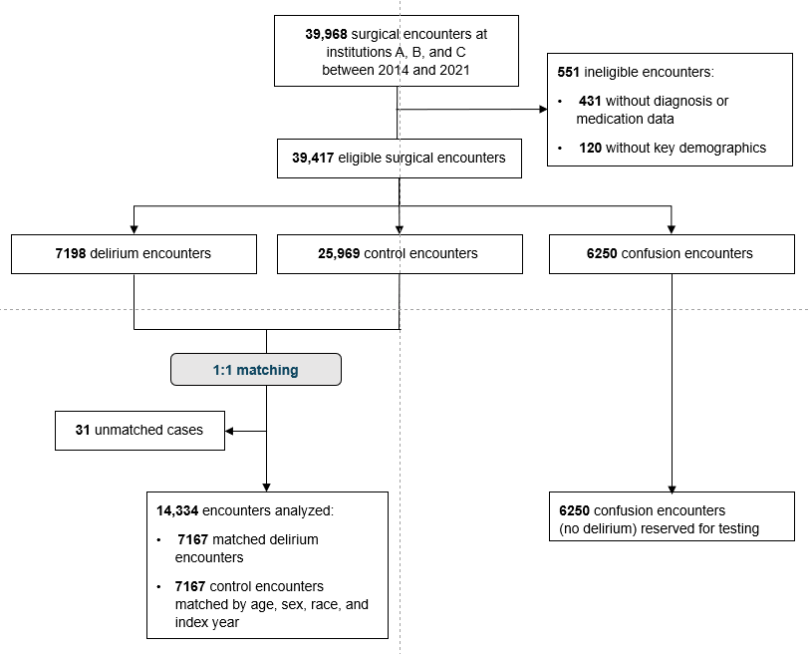
Patient inclusion flow diagram.

Figure 1 depicts the workflow used for model development, internal validation, and external validation for the model trained on data from institution A. Between 2014 and 2022, at the 3 institutions of interest, there were 39,968 surgical visits for 30,131 unique patients aged 50 years and older. Of the identified visits, 431 (1.4%) were excluded for not having any previous diagnosis or medication order data, and 120 (0.4%) were excluded for missing sex, race, or the ASA class. The 6250 (20.7%) visits with nurse-documented confusion (but no delirium) were excluded from the training and holdout sets but reserved for later analyses. After matching, the final analytic sample included 7167 (23.8%) delirium cases and 7167 (23.8%) matched controls (Figure 2).

Pooling across institutions, the median age was 68 (IQR 61-76) years, and most patients were male (n=7412, 51.7%), White (n=12,276, 85.6%), and had public insurance (n=11,523, 80.4%). The most common surgical specialty was general surgery (n=3600, 25.1%), and 11.5% (n=1644) of operations were classified as emergencies (Table 1 and Table S4 in Multimedia Appendix 1).

As shown in Table 2, the 3 most common comorbidities in the general cohort were hypertension (n=9998, 69.8%), diabetes (n=5189, 36.2%), and nonmetastatic cancer (n=5222, 29.6%). Delirium cases differed from controls in several respects. Delirium cases had a greater comorbidity burden than controls and were more likely to have previous delirium (Table 2 and Table S5 in Multimedia Appendix 1).

Of the 6250 (20.7%) visits with nurse-documented confusion but without delirium, 3185 (51%) belonged to institution A, 1328 (21.2%) to institution B, and 1737 (27.8%) to institution C. Patients with confusion were more likely to have had delirium in the past year than controls but less likely than cases. Their comorbidity burden also fell in between that of cases and controls (Tables S6 and S7 in Multimedia Appendix 1).

**Table 1:** Characteristics of delirium cases and controls by institution.

Variables^a^			Institution A				Institution B				Institution C		
		Controls (n=3739)		Cases (n=3739)		Controls (n=1928)		Cases (n=1928)		Controls (n=1500)		Cases (n=1500)	
Age (years), median (IQR)			68 (61-76)		68 (61-76)		66 (59-73)		66 (59-73)		72 (63-80)		72 (63-80)
Sex: female, n (%)			1840 (49.2)		1840 (49.2)		861 (44.7)		861 (44.7)		760 (50.7)		760 (50.7)
**Race, n (%)**													
	Asian	12 (0.3)		12 (0.3)		13 (0.7)		13 (0.7)		1 (0.1)		1 (0.1)	
	Black	758 (20.3)		758 (20.3)		162 (8.4)		162 (8.4)		59 (3.9)		59 (3.9)	
	Other	4 (0.1)		4 (0.1)		3 (0.2)		3 (0.2)		4 (0.3)		4 (0.3)	
	White	2959 (79.1)		2959 (79.1)		1747 (90.6)		1747 (90.6)		1432 (95.5)		1432 (95.5)	
	Unknown	6 (0.2)		6 (0.2)		3 (0.2)		3 (0.2)		4 (0.3)		4 (0.3)	
**Insurance, n (%)**													
	Private	857 (22.9)		572 (15.3)		547 (28.4)		391 (20.3)		239 (15.9)		124 (8.3)	
	Public	2861 (76.5)		3137 (83.9)		1376 (71.4)		1530 (79.4)		1253 (83.5)		1366 (91.1)	
	Uninsured	21 (0.6)		30 (0.8)		5 (0.3)		7 (0.4)		8 (0.5)		10 (0.7)	
BMI, median (IQR)			28.5 (24.3-33.7)		27.5 (23.1-32.7)		27.2 (23.2-32.0)		27.0 (22.7-32.0)		28.0 (23.9-33.6)		27.2 (22.9-33.2)
**Smoking status, n (%)**													
	Current	505 (13.5)		561 (15.0)		173 (9.0)		263 (13.6)		213 (14.2)		280 (18.7)	
	Former	1609 (43.0)		1805 (48.3)		799 (41.4)		901 (46.7)		624 (41.6)		689 (45.9)	
	Never	1625 (43.5)		1373 (36.7)		956 (49.6)		764 (39.6)		663 (44.2)		531 (35.4)	
**ASA^b^ class, n (%)**													
	1-2	421 (11.3)		143 (3.8)		126 (6.5)		37 (1.9)		250 (16.7)		81 (5.4)	
	3-4	3102 (83.0)		2875 (76.9)		1722 (89.3)		1649 (85.5)		1132 (75.5)		1152 (76.8)	
	5 or E	216 (5.8)		721 (19.3)		80 (4.1)		242 (12.6)		118 (7.9)		267 (17.8)	
**Surgical specialty, n (%)**													
	Cardiothoracic (CT)	536 (14.3)		577 (15.4)		183 (9.5)		160 (8.3)		72 (4.8)		142 (9.5)	
	Ears, nose, and throat (ENT)	48 (1.3)		80 (2.1)		76 (3.9)		98 (5.1)		17 (1.1)		77 (5.1)	
	General	498 (13.3)		490 (13.1)		952 (49.4)		981 (50.9)		309 (20.6)		370 (24.7)	
	Multiple	97 (2.6)		614 (16.4)		78 (4.0)		322 (16.7)		15 (1.0)		74 (4.9)	
	Neurology	666 (17.8)		672 (18.0)		3 (0.2)		10 (0.5)		169 (11.3)		128 (8.5)	
	Orthopedics	907 (24.3)		620 (16.6)		103 (5.3)		68 (3.5)		560 (37.3)		370 (24.7)	
	Other	28 (0.7)		28 (0.7)		57 (3.0)		61 (3.2)		11 (0.7)		22 (1.5)	
	Plastic surgery	165 (4.4)		111 (3.0)		31 (1.6)		17 (0.9)		77 (5.1)		95 (6.3)	
	Urology/gynecology	276 (7.4)		172 (4.6)		440 (22.8)		209 (10.8)		153 (10.2)		131 (8.7)	
	Vascular	518 (13.9)		375 (10.0)		5 (0.3)		2 (0.1)		117 (7.8)		91 (6.1)	

^a^Continuous variables are summarized as the median (IQR) and categorical variables as n (%).

^b^ASA: American Society of Anesthesiologists.

**Table 2:** Clinical characteristics of cases and controls by institution.

Variable^a^	Institution A		Institution B		Institution C	
	Controls (n=3739)	Cases (n=3739)	Controls (n=1928)	Cases (n=1928)	Controls (n=1500)	Cases (n=1500)
ECI^b^ score, median (IQR)	5 (0-13)	8 (2-18)	9 (4-17)	13 (5-22)	5 (0-12)	9 (2-18)
Number of ICD^c^ codes, median (IQR)	21 (12-33)	24 (12-40)	21 (11-34)	26 (13-41)	17 (80-29)	22 (11-38)
Congestive heart failure (CHF), n (%)	713 (19.1)	1040 (27.8)	203 (10.5)	304 (15.8)	267 (17.8)	445 (29.7)
Arrhythmia, n (%)	969 (25.9)	1203 (32.2)	397 (20.6)	485 (25.2)	393 (26.2)	471 (31.4)
Valvular disease, n (%)	639 (17.1)	724 (19.4)	148 (7.7)	188 (9.8)	115 (7.7)	178 (11.9)
Peripheral vascular disorder (PVD), n (%)	977 (26.1)	1138 (30.4)	217 (11.3)	259 (13.4)	316 (21.1)	378 (25.2)
Hypertension, n (%)	2767 (74.0)	2696 (72.1)	1217 (63.1)	1255 (65.1)	997 (66.5)	1066 (71.1)
Chronic obstructive pulmonary disorder (COPD), n (%)	962 (25.7)	1227 (32.8)	444 (23.0)	506 (26.2)	398 (26.5)	542 (36.1)
Diabetes, n (%)	1295 (34.6)	1502 (40.2)	558 (28.9)	717 (37.2)	481 (32.1)	636 (42.4)
Hypothyroidism, n (%)	659 (17.6)	630 (16.8)	347 (18.0)	339 (17.6)	249 (16.6)	310 (20.7)
Renal failure, n (%)	891 (23.8)	1198 (32.0)	506 (26.2)	639 (33.1)	336 (22.4)	474 (31.6)
Liver disease, n (%)	266 (7.1)	336 (9.0)	415 (21.5)	573 (29.7)	73 (4.9)	129 (8.6)
Lymphoma, n (%)	69 (1.8)	85 (2.3)	75 (3.9)	71 (3.7)	33 (2.2)	26 (1.7)
Cancer, n (%)	986 (26.4)	1040 (27.8)	1273 (66.0)	1184 (61.4)	339 (22.6)	400 (26.7)
Coagulopathy, n (%)	264 (7.1)	393 (10.5)	164 (8.5)	336 (17.4)	100 (6.7)	153 (10.2)
Obesity, n (%)	720 (19.3)	758 (20.3)	303 (15.7)	372 (19.3)	333 (22.2)	374 (24.9)
Weight loss, n (%)	240 (6.4)	371 (9.9)	220 (11.4)	349 (18.1)	76 (5.1)	173 (11.5)
Fluid/electrolyte disorders, n (%)	761 (20.4)	1171 (31.3)	440 (22.8)	716 (37.1)	334 (22.3)	543 (36.2)
Deficiency anemia, n (%)	460 (12.3)	659 (17.6)	244 (12.7)	335 (17.4)	211 (14.1)	296 (19.7)
Alcohol abuse, n (%)	135 (3.6)	219 (5.9)	67 (3.5)	129 (6.7)	30 (2.0)	74 (4.9)
Drug abuse, n (%)	171 (4.6)	213 (5.7)	58 (3.0)	80 (4.1)	42 (2.8)	72 (4.8)
Psychoses, n (%)	20 (0.5)	84 (2.2)	13 (0.7)	34 (1.8)	12 (0.8)	38 (2.5)
Depression, n (%)	905 (24.2)	1022 (27.3)	343 (17.8)	514 (26.7)	275 (18.3)	406 (27.1)
CVD^d^, n (%)	527 (14.1)	668 (17.9)	111 (5.8)	141 (7.3)	142 (9.5)	231 (15.4)
Previous TBI^e^, n (%)	35 (0.9)	74 (2.0)	12 (0.6)	19 (1.0)	17 (1.1)	23 (1.5)
Sensory impairment, n (%)	212 (5.7)	203 (5.4)	81 (4.2)	91 (4.7)	75 (5.0)	118 (7.9)
Previous delirium, n (%)	215 (5.8)	615 (16.4)	103 (5.3)	304 (15.8)	85 (5.7)	278 (18.5)

^a^Continuous variables are summarized as the median (IQR) and categorical variables as n (%).

^b^ECI: Elixhauser comorbidity index.

^c^ICD: *International Classification of Diseases.*

^d^CVD: cerebrovascular disease.

^e^TBI: traumatic brain injury.

### Model Evaluation

XGB had the highest AUROC out of the 4 candidate classifiers (AUROC=0.79), followed by the neural network (AUROC=0.78), the random forest (AUROC=0.78), and logistic regression (AUROC=0.72). Based on this AUROC evaluation, the XGB model was retained for further analysis. For institution A, the training set included 5234 visits (n=2617, 50%, cases and n=2617, 50%, controls) and the holdout set included 1503 visits (n=752, 50%, cases and n=751, 50%, controls). For institution B, the training and holdout data sets included 2699 visits (n=1350, 50%, cases and n=1349, 50%, controls) and 775 visits (n=387, 49.9%, cases and n=388, 50.1%, controls), respectively. The training and holdout data sets for institution C included 2100 visits (n=1050, 50%, cases and n=1050, 50%, controls) and 603 visits (n=302, 50.1%, cases and n=301, 49.9%, controls), respectively.

The models trained on institution A (ie, XGB_A_) had the best performance, achieving AUROCs of 0.77-0.79 on institution A holdout data and 0.68-0.74 when externally validated on data from institutions B and C. Models trained on institution B (ie, XGB_B_) were the least robust, achieving a maximum AUROC of 0.71 on holdout data from institution B and 0.72-0.74 when externally validated on data from institutions A and C. Models trained on institution C (ie, XGB_C_) performed better than XGB_B_ but worse than XGB_A_, with a maximum AUROC of 0.77 on holdout data from institution C and 0.64-0.75 when externally validated on data from institutions A and B (Table 3).

**Table 3:** XGB^a^ model performance metrics^b^ by surveillance period and holdout data.

Surveillance period, models, and institutions			AUROC^c^, mean (SD)		Sensitivity, mean (SD)		Specificity, mean (SD)		PPV^d^, mean (SD)		NPV^e^, mean (SD)
**1 year, XGB_A_**											
	Institution A	0.79 (0.01)		0.70 (0.02)		0.72 (0.02)		0.72 (0.02)		0.71 (0.02)	
	Institution B	0.69 (0.02)		0.49 (0.03)		0.78 (0.02)		0.69 (0.03)		0.61 (0.02)	
	Institution C	0.74 (0.02)		0.70 (0.03)		0.66 (0.03)		0.67 (0.03)		0.69 (0.03)	
**1 year, XGB_B_**											
	Institution A	0.74 (0.01)		0.76 (0.02)		0.57 (0.02)		0.64 (0.02)		0.70 (0.02)	
	Institution B	0.71 (0.02)		0.57 (0.03)		0.75 (0.02)		0.69 (0.03)		0.64 (0.02)	
	Institution C	0.73 (0.02)		0.65 (0.03)		0.68 (0.03)		0.67 (0.03)		0.66 (0.03)	
**1 year, XGC_C_**											
	Institution A	0.75 (0.01)		0.75 (0.02)		0.60 (0.02)		0.66 (0.02)		0.71 (0.02)	
	Institution B	0.69 (0.02)		0.47 (0.03)		0.77 (0.02)		0.67 (0.03)		0.59 (0.02)	
	Institution C	0.77 (0.02)		0.66 (0.03)		0.69 (0.03)		0.69 (0.03)		0.67 (0.03)	
**6 months, XGB_A_**											
	Institution A	0.78 (0.01)		0.56 (0.03)		0.73 (0.02)		0.67 (0.03)		0.62 (0.02)	
	Institution B	0.68 (0.02)		0.45 (0.03)		0.79 (0.02)		0.68 (0.03)		0.59 (0.02)	
	Institution C	0.74 (0.02)		0.67 (0.03)		0.66 (0.03)		0.67 (0.03)		0.67 (0.03)	
**6 months, XGB_B_**											
	Institution A	0.73 (0.01)		0.78 (0.02)		0.54 (0.02)		0.63 (0.02)		0.71 (0.02)	
	Institution B	0.71 (0.02)		0.56 (0.03)		0.73 (0.02)		0.67 (0.03)		0.62 (0.02)	
	Institution C	0.74 (0.02)		0.66 (0.03)		0.68 (0.03)		0.68 (0.03)		0.67 (0.03)	
**6 months, XGC_C_**											
	Institution A	0.73 (0.01)		0.76 (0.02)		0.55 (0.02)		0.63 (0.02)		0.70 (0.02)	
	Institution B	0.65 (0.02)		0.52 (0.03)		0.70 (0.02)		0.64 (0.03)		0.60 (0.02)	
	Institution C	0.76 (0.02)		0.71 (0.03)		0.66 (0.03)		0.68 (0.03)		0.69 (0.03)	
**3 months, XGB_A_**											
	Institution A	0.77 (0.01)		0.70 (0.02)		0.70 (0.02)		0.70 (0.02)		0.70 (0.02)	
	Institution B	0.69 (0.02)		0.47 (0.03)		0.78 (0.02)		0.68 (0.03)		0.60 (0.02)	
	Institution C	0.74 (0.02)		0.68 (0.03)		0.67 (0.03)		0.67 (0.03)		0.68 (0.03)	
**3 months, XGB_B_**											
	Institution A	0.72 (0.01)		0.75 (0.02)		0.55 (0.02)		0.63 (0.02)		0.69 (0.02)	
	Institution B	0.70 (0.02)		0.56 (0.03)		0.74 (0.02)		0.68 (0.03)		0.63 (0.02)	
	Institution C	0.74 (0.02)		0.65 (0.03)		0.68 (0.03)		0.67 (0.03)		0.66(0.03)	
**3 months, XGC_C_**											
	Institution A	0.73 (0.01)		0.75 (0.02)		0.57 (0.02)		0.64 (0.02)		0.70 (0.02)	
	Institution B	0.64 (0.02)		0.50 (0.03)		0.71 (0.02)		0.63 (0.03)		0.58 (0.02)	
	Institution C	0.76 (0.02)		0.73 (0.03)		0.64 (0.03)		0.67 (0.03)		0.70 (0.03)	

^a^XGB: extreme gradient boosting.

^b^Mean (SD) metrics presented were obtained using bootstrap resampling on the held-out patients from institutions A, B, and C.

^c^AUROC: area under the receiver operating curve.

^d^PPV: positive predictive value.

^e^NPV: negative predictive value.

Performance became marginally worse with shorter surveillance periods. All models were relatively well calibrated (Figures S1-S3 in Multimedia Appendix 1). The top 5 most important features for XGB_A_, XGB_B_, and XGB_C_ by evaluation data set and surveillance period are presented in Table 4 and Tables S8-S9 in Multimedia Appendix 1. The ASA class was frequently the most important predictor.

Across all surveillance periods, the models predicted between 40% and 60% of the patients with confusion as cases or controls (Table S10 in Multimedia Appendix 1).

**Table 4:** Top 5 most influential variables used by XGB^a^ models (1-year surveillance period).^b^

Model and rank			Holdout data			
			Institution A		Institution B	Institution C
**XGB_A_**						
	1	ASA^c^ class		ASA class		ASA class
	2	ICD^d^ group: Z00-Z13^e^		ICD group: Z00-Z13		ICD group: Z00-Z13
	3	Multispecialty surgery		Multispecialty surgery		Service: hospitalist^f^
	4	Service: hospitalist		Service: hospitalist		Multispecialty surgery
	5	Emergency surgery		Previous delirium		Emergency surgery
**XGB_B_**						
	1	ASA class		ASA class		ASA class
	2	Multispecialty surgery		Multispecialty surgery		Multispecialty surgery
	3	Previous delirium		Previous delirium		Previous delirium
	4	BMI		Urology/gynecology surgery		Service: orthopedics^g^
	5	Emergency surgery		BMI		BMI
**XGB_C_**						
	1	ASA class		ASA class		ASA class
	2	Service: hospitalist		Service: hospitalist		Service: orthopedics
	3	Service: orthopedics		Service: orthopedics		Service: hospitalist
	4	Previous delirium		Previous delirium		Previous delirium
	5	Multispecialty surgery		Multispecialty surgery		ICD group: Z77-Z99^h^

^a^XGB: extreme gradient boosting.

^b^Feature importance measured using Shapley Additive Explanation (SHAP) values. XGB_A_, XGB_B_, and XGB_C_ were trained on data from institutions A, B, and C, respectively.

^c^ASA: American Society of Anesthesiologists.

^d^ICD: *International Classification of Diseases.*

^e^ICD group Z00-Z13: persons encountering health services for examinations.

^f^Admitted to hospitalist service.

^g^Admitted to orthopedics service.

^h^ICD group Z77-Z99: persons with potential health hazards related to family and personal history and certain conditions influencing health status.

## Discussion

### Principal Findings

We developed and externally validated 3 models to predict POD with routine EHR data available at the time of hospital admission. In our experiments, XGB outperformed all other classifiers and demonstrated good discriminative ability on holdout data, achieving a maximum AUROC of 0.79. Generalizability varied by model and the institution used for external validation.

Our models demonstrated good predictive accuracy, with XGB_A_ outperforming XGB_B_ and XGB_C_ across all surveillance periods. Interestingly, longer surveillance periods did not appear to significantly benefit model performance. This is likely because the most important features were surgery-related variables, which were fixed across all surveillance durations. Additionally, surveillance duration did not impact how the models classified patients with confusion but no delirium (ie, potential subsyndromal delirium); approximately half were predicted to be cases, and the other half were predicted to be controls, regardless of the surveillance period. Given that subsyndromal delirium is thought to be on the spectrum between healthy controls and delirium [38], it was expected that the models would have trouble classifying those patients.

Generalizability varied by model and institution. XGB_A_ performed relatively well when externally validated using data from institution C, as did XGB_C_ when validated using data from institution A. However, the AUROCs for both models decreased substantially when validated on data from institution B. In contrast, XGB_B_ had higher AUROCs when externally validated on institutions A and C than it did on holdout data from the same institution it was trained on. We hypothesize that the observed variation in performance could be due to institution B having a substantially different patient population than institutions A and C. Institutions A and C are trauma centers that perform a comparatively large number of orthopedic surgeries, and their populations have fewer comorbidities. Institution A also cares for complex vascular and cardiac patients, while the other 2 institutions generally do not. Conversely, institution B is not a trauma center and performs mostly general and urologic/gynecologic surgeries. It also largely services frail, high-acuity patients with chronic illnesses, and the general surgical complexity is higher. The comparatively low AUROC of XGB_B_ could reflect the model having difficulty discriminating between cases and controls, because it was trained on patients who were more ill, regardless of delirium status. These results highlight the importance of selecting an appropriate training population when a generalizable prediction model is desired; if a hospital has a patient population that differs significantly from the training data set, a localized model may be needed, even within the same hospital system.

The ASA class, a subjective measure of a patient’s physiologic status [39], was frequently the most important feature. This supports previous literature linking a higher ASA class to a greater risk of POD [40]. The Elixhauser comorbidity index (ECI) did not appear in the list of top features despite the strong association of comorbidities with delirium, possibly because the ASA class summarizes health information beyond mortality risk and additionally identifies emergency cases. However, the subjectivity of the ASA class [41] may harm model generalizability compared to more objective measures, such as comorbidity scores. Other surgical variables, including admitting service and surgical specialty, were frequently among the top 5 features. Notably, both these variables have been associated with an increased risk of POD, particularly surgical specialty [6]. Multispecialty surgery was particularly important across models, suggesting that surgical complexity may be an important risk factor for delirium. The type of admitting service and individual surgical specialties that were most predictive differed by model, potentially because the distributions were different between institutions. For example, urologic/gynecologic surgery was frequently a top predictor in XGB_B_ models but not in others. This could be because proportionally more controls had that type of surgery than cases at institution B but not at institutions A and C. Reducing the cardinality of these variables is likely to improve generalizability but potentially at the cost of reduced discriminative ability. For XGB_A_, the number of ICD codes belonging to ICD-10 group Z00-Z13 (“persons encountering health services for examinations”) was a top feature, and higher values negatively influenced model predictions. This may be because this ICD group captures routine health examinations, which are often undertaken by healthier individuals. The fact that the top features are supported by the literature suggests that the models are clinically explainable.

Several delirium prediction models have been developed, reporting AUROCs ranging from 0.56 to 0.94 [42]. The models with the highest AUROCs focus on specific patient subsets (ie, ICU patients, cardiac surgery) and include variables collected during the hospital stay, such as the APACHE score (which must be calculated), surgery duration (often not reliably recorded), and inpatient laboratory values. In-hospital variables may, indeed, be the strongest predictors of delirium and explain why our model failed to outperform previous ones; however, they were intentionally excluded from this study as that would preclude our models from being used at the time of hospitalization. Fewer models have been developed that are both her based and intended to be used at or shortly after admission. In their 2022 paper, Bishara et al [14] developed a POD prediction model for the general surgical population using different machine learning approaches and preoperative EHR data. They found that an XGB model outperforms other classifiers, similar to our findings, and reported an internal validation AUROC of 0.85 [14]. In contrast to our study, matching was not performed, and patients with dementia were included in the study population. Fifty-nine variables derived from inpatient (but preoperative) nursing assessments were also included as predictors. Some of these assessments (eg, Braden Scale score [43]) captured patients’ functional status, which is highly correlated to delirium [5,6] and may explain why their model had a higher AUROC. Wong et al [44] developed a model to predict delirium in a general inpatient population without known cognitive impairment using an XGB model and reported an AUROC of 0.86. Their model used 796 features collected within 24 hours of admission and included inpatient neurologic examination data, which were highly predictive of delirium. These factors could explain, at least in part, the difference in performance between these previous models and our models.

In summary, our findings suggest that a machine learning model trained on routine EHR data can achieve clinically useful accuracy when predicting POD. Unlike previous models, the models presented in this study can be used to make predictions at the time of hospital admission, which could quickly inform preventive and resource-planning efforts. The models were also externally validated, providing critical information about generalizability when using a limited set of prehospital and surgery variables. These models can be readily integrated into EHR systems to provide a scalable, automated prescreening tool to flag patients who are at risk of developing POD and would benefit from targeted preventative measures.

### Strengths and Limitations

Our study has several strengths. First, we used both the CAM method and ICD codes to maximize case identification; because delirium ICD codes are extremely specific but less sensitive [45], false negatives are unlikely. Second, we compared different surveillance periods to determine how surveillance duration influences accuracy. Third, we examined how the models classify patients with confusion but no delirium, which could potentially capture subsyndromal delirium. Finally, we trained our models on data from 3 different institutions and externally validated them against each other to determine their transportability.

This study also has several limitations. Although we attempted to maximize delirium detection by using both the CAM method and ICD codes, a small number of patients did not have any CAM data available. As mentioned previously, delirium ICD codes tend to have high specificity but lower sensitivity [45], so some cases may have been missed. Patients were intentionally matched on age, sex, and race to limit biases related to these variables; however, discriminative ability was likely reduced as a result. Because patients with preexisting dementia or confusion during the inpatient visit (but no documented delirium) were excluded, the models may not generalize well to those types of patients. However, we chose to exclude those patients because their high risk of delirium was evident; our models focused on patients with a less clear delirium risk, which could partially explain the lower performance compared to previous models. Finally, although the models were externally validated, the hospitals were within the same health care system, which may present more optimistic generalizability relative to uses of the models in outside systems.

### Conclusion

Routine EHR data can be used for early delirium prediction in a diverse cohort of surgery patients without dementia. Although our models slightly underperformed relative to some of the previously published classifiers that use inpatient data, our routine EHR-based models serve a distinct purpose of enabling predictions at the time of admission, while being highly scalable. Generalizability varied depending on the training data, so institution-specific models may be necessary when using only a limited set of preadmission and surgery variables with distributions that substantially differ between institutions. The proposed models could be used in clinical practice as an automated prescreening tool for the early identification of high-risk patients, enabling clinicians to immediately adjust their care strategies and inform targeted delirium prevention measures and resource planning.

## Appendix

Multimedia Appendix 1 Calibration curves for XGB; ICD codes for preexisting Alzheimer’s disease, related dementias, delirium, and additional variables; sociodemographic and surgical characteristics of patients and controls; clinical characteristics of patients and controls; XGB model predictions for confusion encounters; and top 5 most influential variables used by XGB models. ICD: International Classification of Diseases; XGB: extreme gradient boosting.

## Abbreviations

ACB: Anticholinergic Cognitive Burden
ACh: anticholinergic
ASA: American Society of Anesthesiologists
ATC: Anatomical Therapeutic Chemical
AUROC: area under the receiver operating characteristic curve
CAM: Confusion Assessment Method
CVD: cerebrovascular disease
ECI: Elixhauser comorbidity index
EHR: electronic health record
ICD: International Classification of Diseases
ICD-9: International Classification of Diseases, Ninth Revision
ICD-10-CM: International Classification of Diseases, Tenth Revision, Clinical Modification
IU: Indiana University
NPV: negative predictive value
POD: postoperative delirium
PPV: positive predictive value
SHAP: Shapley Additive Explanation
TBI: traumatic brain injury
XGB: extreme gradient boosting
