# Supplementary material for: Development and Validation of a Routine Electronic Health Record-Based Delirium Prediction Model for Surgical Patients Without Dementia: Retrospective Case-Control Study
Source: JMIR Perioper Med. 2025 Jan 9;8:e59422. doi: 10.2196/59422 (PMC11757977; doi:10.2196/59422)
Supplement: Multimedia Appendix 1 [file periop_v8i1e59422_app1.docx]

Figure S1. Calibration curves for XGBa by surveillance period


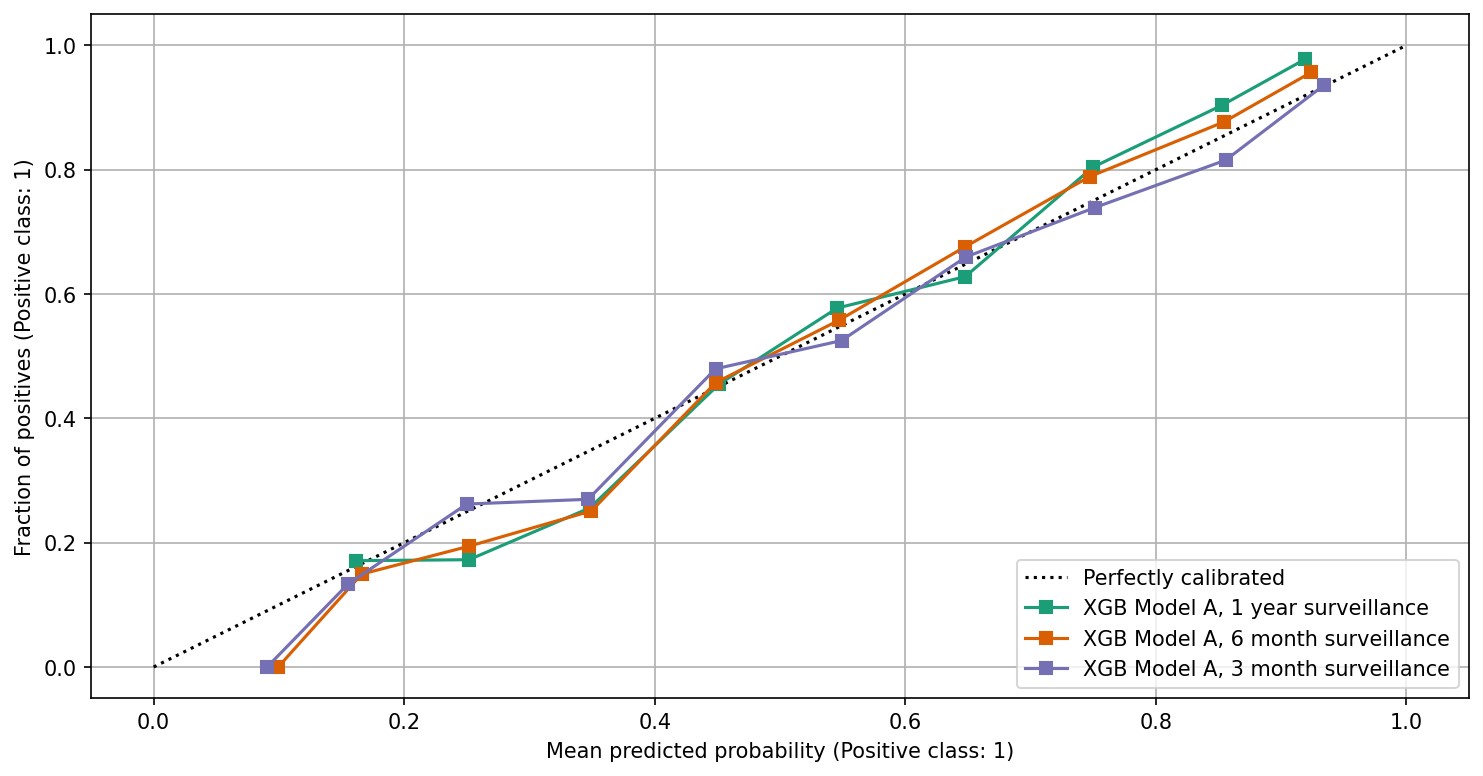


Figure S2. Calibration curves for XGBb for each surveillance period


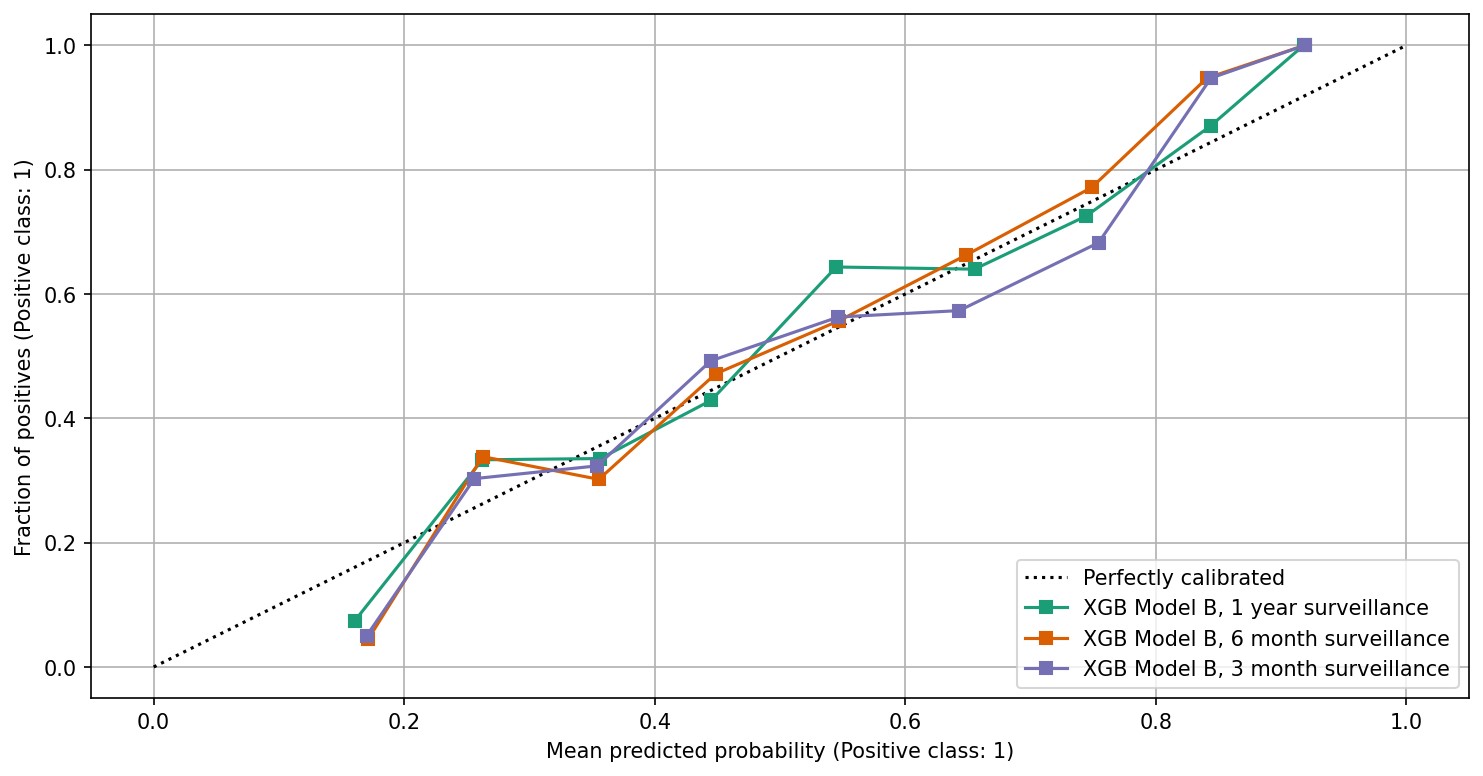


Figure S3. Calibration curves for XGBc for each surveillance period


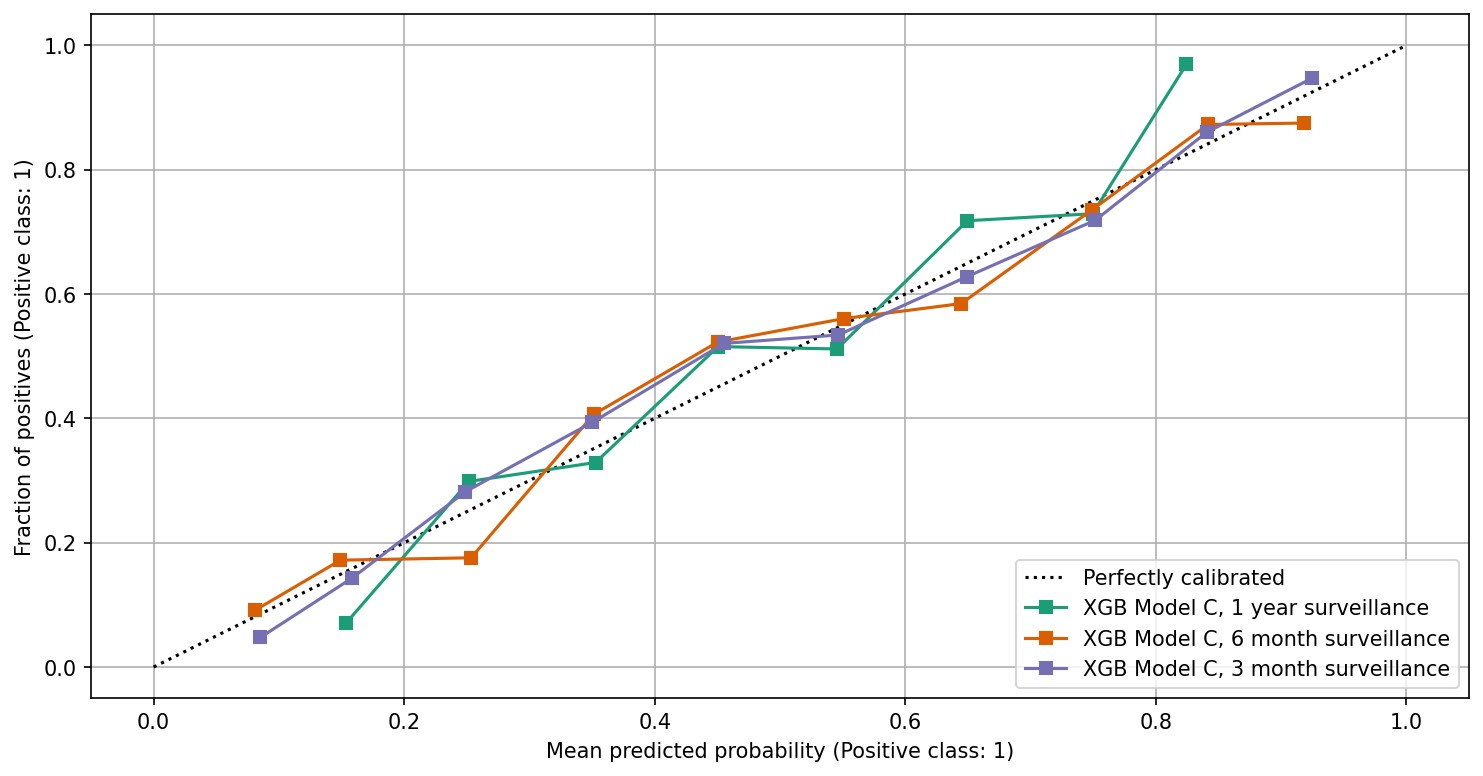


| Table S1. ICD codes and medications used to identify pre-existing alzheimer’s disease and related dementias | | |
| --- | --- | --- |
| **ICD Codes for Alzheimer’s Disease and Related Dementias** | | |
| **ICD Code** | **Type** | **Definition** |
| 290 | ICD-9 | Dementias |
| 290.0 | ICD-9 | Senile dementia, uncomplicated |
| 290.1 | ICD-9 | Presenile dementia |
| 290.10 | ICD-9 | Pre-senile dementia, uncomplicated |
| 290.11 | ICD-9 | Pre-senile dementia with delirium |
| 290.12 | ICD-9 | Pre-senile dementia with delusional features |
| 290.13 | ICD-9 | Pre-senile dementia with depressive features |
| 290.2 | ICD-9 | Senile dementia with delusional or depressive features |
| 290.20 | ICD-9 | Senile dementia with delusional features |
| 290.21 | ICD-9 | Senile dementia with depressive features |
| 290.3 | ICD-9 | Senile dementia with delirium |
| 290.4 | ICD-9 | Vascular dementia |
| 290.40 | ICD-9 | Vascular dementia, uncomplicated |
| 290.41 | ICD-9 | Vascular dementia with delirium |
| 290.42 | ICD-9 | Vascular dementia with delusions |
| 290.43 | ICD-9 | Vascular dementia with depressed mood |
| 290.8 | ICD-9 | Other specified senile psychotic conditions |
| 290.9 | ICD-9 | Unspecified senile psychotic condition |
| 291.2 | ICD-9 | Alcohol induced persisting dementia |
| 292.82 | ICD-9 | Drug induced persisting dementia |
| 294.1 | ICD-9 | Dementia in conditions classified elsewhere |
| 294.10 | ICD-9 | Dementia in conditions classified elsewhere with behavior |
| 294.11 | ICD-9 | Dementia in conditions classified elsewhere with behavioral |
| 294.2 | ICD-9 | Dementia, unspecified |
| 294.2 | ICD-9 | Dementia, unspecified without behavioral disturbance |
| 294.21 | ICD-9 | Dementia, unspecified with behavioral disturbance |
| 331.0 | ICD-9 | Alzheimer’s disease |
| 331.1 | ICD-9 | Frontotemporal dementia |
| 331.11 | ICD-9 | Pick’s disease |
| 331.19 | ICD-9 | Other frontotemporal dementia |
| 331.2 | ICD-9 | Senile degeneration of brain |
| 331.82 | ICD-9 | Dementia with Lewy bodies |
| 331.9 | ICD-9 | Degenerative disease of nervous system, unspecified |
| A81.00 | ICD-10 | Creutzfeldt-Jakob disease or syndrome (with dementia) |
| F01 | ICD-10 | Vascular dementia |
| F01.50 | ICD-10 | Vascular dementia without behavioral disturbance |
| F01.51 | ICD-10 | Vascular dementia with behavioral disturbance |
| F02 | ICD-10 | Dementia in other diseases classified elsewhere |
| F02.8 | ICD-10 | Dementia in other diseases classified elsewhere |
| F02.80 | ICD-10 | Dementia in OTH disease classified elsewhere without behavioral disturbance |
| F02.81 | ICD-10 | Dementia in OTH disease classified elsewhere with behavioral disturbance |
| F03 | ICD-10 | Unspecified dementia |
| F03.9 | ICD-10 | Unspecified dementia |
| F03.90 | ICD-10 | Unspecified dementia without behavioral disturbance |
| F03.91 | ICD-10 | Unspecified dementia with behavioral disturbance |
| F10.27 | ICD-10 | Alcohol dependence with alcohol-induced persisting dementia |
| F10.97 | ICD-10 | Alcohol use, unspecified with alcohol induced persisting dementia |
| F13.27 | ICD-10 | Sedative, hypnotic or anxiolytic dependence with sedative, hypnotic or anxiolytic-induced persisting dementia |
| F13.97 | ICD-10 | Sedative, hypnotic or anxiolytic use, unspecified with sedative, hypnotic or anxiolytic-induced persisting dementia |
| F18.27 | ICD-10 | Inhalant dependence with inhalant-induced dementia |
| F18.97 | ICD-10 | Inhalant use, unspecified with inhalant-induced persisting dementia |
| F19.27 | ICD-10 | Other psychoactive substance dependence with psychoactive substance-induced persisting dementia |
| F19.97 | ICD-10 | Other psychoactive substance use, unspecified with psychoactive substance-induced persisting dementia |
| G30 | ICD-10 | Alzheimer’s disease |
| G30.0 | ICD-10 | Alzheimer’s disease with early onset |
| G30.1 | ICD-10 | Alzheimer's disease with late onset |
| G30.8 | ICD-10 | Other Alzheimer's disease |
| G30.9 | ICD-10 | Alzheimer’s disease, unspecified |
| G31.0 | ICD-10 | Frontotemporal dementia |
| G31.09 | ICD-10 | Other frontotemporal dementia |
| G31.1 | ICD-10 | Senile degeneration of brain, not elsewhere classified |
| G31.83 | ICD-10 | Dementia with Lewy bodies |
| G31.9 | ICD-10 | Degenerative disease of nervous system, unspecified |
| **Anti-Dementia Medications** | | |
| Donepezil, Galantamine , Rivastigmine, Tacrine, Memantine, Donepezil + memantine | | |

| Table S2. ICD Codes for Delirium | | |
| --- | --- | --- |
| **ICD Code** | **Type** | **Definition** |
| 348.3 | ICD-9 | Encephalopathy, unspecified |
| 293 | ICD-9 | Delirium due to conditions classified elsewhere |
| 780.97 | ICD-9 | Altered mental status |
| 291 | ICD-9 | Alcohol withdrawal delirium |
| 780.1 | ICD-9 | Hallucinations |
| 780.09 | ICD-9 | Other alteration of consciousness |
| 292 | ICD-9 | Drug withdrawal |
| 290.11 | ICD-9 | Presenile dementia with delirium |
| 290.3 | ICD-9 | Senile dementia with delirium |
| 290.41 | ICD-9 | Vascular dementia, with delirium |
| 292.81 | ICD-9 | Drug-induced delirium |
| 293.1 | ICD-9 | Subacute delirium |
| 293.81 | ICD-9 | Psychotic disorder with delusions in conditions classified elsewhere |
| 293.82 | ICD-9 | Psychotic disorder with hallucinations in conditions classified elsewhere |
| 293.89 | ICD-9 | Other specified transient mental disorders due to conditions classified elsewhere |
| 293.9 | ICD-9 | Unspecified transient mental disorder in conditions classified elsewhere |
| 290.8 | ICD-9 | Other specified senile psychotic conditions |
| 290.9 | ICD-9 | Unspecified senile psychotic condition |
| 292.11 | ICD-9 | Drug-induced psychotic disorder with delusions |
| 292.12 | ICD-9 | Drug-induced psychotic disorder with hallucinations |
| 292.2 | ICD-9 | Pathological drug intoxication |
| 348.31 | ICD-9 | Metabolic encephalopathy |
| 348.39 | ICD-9 | Other encephalopathy |
| 349.82 | ICD-9 | Toxic encephalopathy |
| 780.02 | ICD-9 | Transient alteration of awareness |
| E939.3 | ICD-9 | Other antipsychotics, neuroleptics, and major tranquilizers causing adverse effects in therapeutic use |
| R41.0 | ICD-10 | Disorientation, unspecified |
| R41.82 | ICD-10 | Altered mental status, unspecified |
| R44.3 | ICD-10 | Hallucinations, unspecified |
| R44.1 | ICD-10 | Visual hallucinations |
| R44.2 | ICD-10 | Other hallucinations |
| R40.4 | ICD-10 | Transient alteration of awareness |
| T43.595A | ICD-10 | Adverse effect of unspecified antipsychotics and neuroleptics, initial encounter |
| R44.0 | ICD-10 | Auditory hallucinations |
| E51.2 | ICD-10 | Wernicke's encephalopathy |
| G93.40 | ICD-10 | Encephalopathy, unspecified |
| A81.2 | ICD-10 | Progressive multifocal leukoencephalopathy |
| F05 | ICD-10 | Delirium due to known physiological condition |
| F10.121 | ICD-10 | Alcohol abuse with intoxication delirium |
| F10.221 | ICD-10 | Alcohol dependence with intoxication delirium |
| F10.231 | ICD-10 | Alcohol dependence with withdrawal delirium |
| F10.921 | ICD-10 | Alcohol use, unspecified with intoxication delirium |
| F11.121 | ICD-10 | Opioid abuse with intoxication delirium |
| F11.221 | ICD-10 | Opioid dependence with intoxication delirium |
| F11.921 | ICD-10 | Opioid use, unspecified with intoxication delirium |
| F12.121 | ICD-10 | Cannabis abuse with intoxication delirium |
| F12.221 | ICD-10 | Cannabis dependence with intoxication delirium |
| F12.921 | ICD-10 | Cannabis use, unspecified with intoxication delirium |
| F13.121 | ICD-10 | Sedative, hypnotic or anxiolytic abuse with intoxication delirium |
| F13.221 | ICD-10 | Sedative, hypnotic or anxiolytic dependence with intoxication delirium |
| F13.231 | ICD-10 | Sedative, hypnotic or anxiolytic dependence with withdrawal delirium |
| F13.921 | ICD-10 | Sedative, hypnotic or anxiolytic use, unspecified with intoxication delirium |
| F13.931 | ICD-10 | Sedative, hypnotic or anxiolytic use, unspecified with withdrawal delirium |
| F14.121 | ICD-10 | Cocaine abuse with intoxication with delirium |
| F14.221 | ICD-10 | Cocaine dependence with intoxication delirium |
| F14.921 | ICD-10 | Cocaine use, unspecified with intoxication delirium |
| F15.121 | ICD-10 | Other stimulant abuse with intoxication delirium |
| F15.221 | ICD-10 | Other stimulant dependence with intoxication delirium |
| F15.921 | ICD-10 | Other stimulant use, unspecified with intoxication delirium |
| F16.121 | ICD-10 | Hallucinogen abuse with intoxication with delirium |
| F16.221 | ICD-10 | Hallucinogen dependence with intoxication with delirium |
| F16.921 | ICD-10 | Hallucinogen use, unspecified with intoxication with delirium |
| F18.121 | ICD-10 | Inhalant abuse with intoxication delirium |
| F18.221 | ICD-10 | Inhalant dependence with intoxication delirium |
| F18.921 | ICD-10 | Inhalant use, unspecified with intoxication with delirium |
| F19.121 | ICD-10 | Other psychoactive substance abuse with intoxication delirium |
| F19.221 | ICD-10 | Other psychoactive substance dependence with intoxication delirium |
| F19.231 | ICD-10 | Other psychoactive substance dependence with withdrawal delirium |
| F19.921 | ICD-10 | Other psychoactive substance use, unspecified with intoxication with delirium |
| F19.931 | ICD-10 | Other psychoactive substance use, unspecified with withdrawal delirium |
| G04.30 | ICD-10 | Acute necrotizing hemorrhagic encephalopathy, unspecified |
| G04.31 | ICD-10 | Post-infectious acute necrotizing hemorrhagic encephalopathy |
| G04.32 | ICD-10 | Post-immunization acute necrotizing hemorrhagic encephalopathy |
| G04.39 | ICD-10 | Other acute necrotizing hemorrhagic encephalopathy |
| G92 | ICD-10 | Toxic encephalopathy |
| G93.41 | ICD-10 | Metabolic encephalopathy |
| G93.49 | ICD-10 | Other encephalopathy |
| I67.3 | ICD-10 | Progressive vascular leukoencephalopathy |
| I67.4 | ICD-10 | Hypertensive encephalopathy |
| I67.83 | ICD-10 | Posterior reversible encephalopathy syndrome |
| J10.81 | ICD-10 | Influenza due to other identified influenza virus with encephalopathy |
| J11.81 | ICD-10 | Influenza due to unidentified influenza virus with encephalopathy |
| P91.60 | ICD-10 | Hypoxic ischemic encephalopathy, unspecified |
| P91.61 | ICD-10 | Mild hypoxic ischemic encephalopathy |
| P91.62 | ICD-10 | Moderate hypoxic ischemic encephalopathy |
| P91.63 | ICD-10 | Severe hypoxic ischemic encephalopathy |
| T43.505D | ICD-10 | Adverse effect of unspecified antipsychotics and neuroleptics, subsequent encounter |

| Table S3. ICD Codes for Additional Variables | | |
| --- | --- | --- |
| **Sensory Impairment** | | |
| **ICD Code** | **Type** | **Definition** |
| 369.x | ICD-9 | Blindness and low vision |
| 389.x | ICD-9 | Hearing loss |
| H54.x | ICD-10 | Blindness and low vision |
| H90.x | ICD-10 | Conductive and sensorineural hearing loss |
| H91.x | ICD-10 | Other unspecified hearing loss |
| **Traumatic Brain Injury** | | |
| **ICD Code** | **Type** | **Definition** |
| 850.x | ICD-9 | Concussion |
| 851.x | ICD-9 | Cerebral laceration and contusion |
| 852.x | ICD-9 | Subarachnoid subdural and extradural hemorrhage following injury |
| 853.x | ICD-9 | Other and unspecified intracranial hemorrhage following injury |
| 854.x | ICD-9 | Intracranial injury of other and unspecified nature |
| S06.x | ICD-10 | Intracranial injury |
| Z87.820 | ICD-10 | Personal history of traumatic brain injury |
| **Cerebrovascular Disease** | | |
| **ICD Code** | **Type** | **Definition** |
| 362.34 | ICD-9 | Transient retinal arterial occlusion |
| 430.x | ICD-9 | Subarachnoid hemorrhage |
| 431.x | ICD-9 | Intracerebral hemorrhage |
| 432.x | ICD-9 | Other and unspecified intracranial hemorrhage |
| 433.x | ICD-9 | Occlusion and stenosis of precerebral arteries |
| 434.x | ICD-9 | Occlusion of cerebral arteries |
| 435.x | ICD-9 | Transient cerebral ischemia |
| 436.x | ICD-9 | Acute, but ill-defined, cerebrovascular disease |
| 437.x | ICD-9 | Other and ill-defined cerebrovascular disease |
| 438.x | ICD-9 | Late effects of cerebrovascular disease |
| G45.x | ICD-10 | Transient cerebral ischemic attacks and related syndromes |
| G46.x | ICD-10 | Vascular syndromes of brain in cerebrovascular diseases |
| H34.0 | ICD-10 | Transient retinal artery occlusion |
| I60.x | ICD-10 | Nontraumatic subarachnoid hemorrhage |
| I61.x | ICD-10 | Nontraumatic intracerebral hemorrhage |
| I62.x | ICD-10 | Other and unspecified nontraumatic intracranial hemorrhage |
| I63.x | ICD-10 | Cerebral infarction |
| I65.x | ICD-10 | Occlusion and stenosis of precerebral arteries, not resulting in cerebral infarction |
| I66.x | ICD-10 | Occlusion and stenosis of cerebral arteries, not resulting in cerebral infarction |
| I67.x | ICD-10 | Other cerebrovascular diseases |
| I68.x | ICD-10 | Cerebrovascular disorders in diseases classified elsewhere |
| I69.x | ICD-10 | Sequelae of cerebrovascular disease |

| Table S4. Sociodemographic and surgical characteristics of delirium cases and controls | | | |
| --- | --- | --- | --- |
| **Variable** | **Controls**  **(N=7167)** | **Cases**  **(N=7167)** | **Total**  **(N=14334)** |
| Age | 68.0 (61.0-76.0) | 68.0 (61.0-76.0) | 68.0 (61.0-76.0) |
| Female | 3461 (48.3%) | 3461 (48.3%) | 6922 (48.3%) |
| Race |  |  |  |
| Asian | 26 (0.4%) | 26 (0.4%) | 52 (0.4%) |
| Black | 979 (13.7%) | 979 (13.7%) | 1958 (13.7%) |
| Other | 11 (0.2%) | 11 (0.2%) | 22 (0.2%) |
| White | 6138 (85.6%) | 6138 (85.6%) | 12276 (85.6%) |
| Unknown | 13 (0.2%) | 13 (0.2%) | 26 (0.2%) |
| Insurance |  |  |  |
| Private | 1643 (22.9%) | 1087 (15.2%) | 2730 (19.0%) |
| Public | 5490 (76.6%) | 6033 (84.2%) | 11523 (80.4%) |
| Uninsured | 34 (0.5%) | 47 (0.7%) | 81 (0.6%) |
| BMI | 28.0 (23.9-33.3) | 27.3 (23.0-32.6) | 27.6 (23.4-33.0) |
| Smoking Status |  |  |  |
| Current | 891 (12.4%) | 1104 (15.4%) | 1995 (13.9%) |
| Former | 3032 (42.3%) | 3395 (47.4%) | 6427 (44.8%) |
| Never | 3244 (45.3%) | 2668 (37.2%) | 5912 (41.2%) |
| ASA class |  |  |  |
| 1-2 | 797 (11.1%) | 261 (3.6%) | 1058 (7.4%) |
| 3-4 | 5956 (83.1%) | 5676 (79.2%) | 11632 (81.1%) |
| 5 or E | 414 (5.8%) | 1230 (17.2%) | 1644 (11.5%) |
| Primary specialty |  |  |  |
| CT | 791 (11.0%) | 879 (12.3%) | 1670 (11.7%) |
| ENT | 141 (2.0%) | 255 (3.6%) | 396 (2.8%) |
| General | 1759 (24.5%) | 1841 (25.7%) | 3600 (25.1%) |
| Multiple | 190 (2.7%) | 1010 (14.1%) | 1200 (8.4%) |
| Neurology | 838 (11.7%) | 810 (11.3%) | 1648 (11.5%) |
| Orthopedics | 1570 (21.9%) | 1058 (14.8%) | 2628 (18.3%) |
| Other | 96 (1.3%) | 111 (1.5%) | 207 (1.4%) |
| Plastics | 273 (3.8%) | 223 (3.1%) | 496 (3.5%) |
| Urogyn | 869 (12.1%) | 512 (7.1%) | 1381 (9.6%) |
| Vascular | 640 (8.9%) | 468 (6.5%) | 1108 (7.7%) |
| Continuous variables are summarized as median (IQR) and categorical variables as n (%). Abbreviations: BMI, body mass index; ASA, American Society of Anesthesiologists; CT, cardiothoracic, ENT, ears, nose, and throat; urogyn = urology gynecology. | | | |

| Table S5. Clinical characteristics of delirium cases and controls | | | |
| --- | --- | --- | --- |
| **Variables** | **Controls (N=7167)** | **Cases (N=7167)** | **Total**  **(N=14334)** |
| ECI Score | 6.00 (0-14.0) | 10.0 (2.00-19.0) | 8.00 (1.00-16.0) |
| Number of ICD codes | 20.0 (11.0-32.0) | 24.0 (12.0-40.0) | 22.0 (12.0-36.0) |
| Comorbidities |  |  |  |
| Congestive heart failure | 1183 (16.5%) | 1789 (25.0%) | 2972 (20.7%) |
| Cardiac arrhythmias | 1759 (24.5%) | 2159 (30.1%) | 3918 (27.3%) |
| Valvular disease | 902 (12.6%) | 1090 (15.2%) | 1992 (13.9%) |
| Pulmonary circulation disorders | 448 (6.3%) | 684 (9.5%) | 1132 (7.9%) |
| Peripheral vascular disorders | 1510 (21.1%) | 1775 (24.8%) | 3285 (22.9%) |
| Hypertension | 4981 (69.5%) | 5017 (70.0%) | 9998 (69.8%) |
| Paralysis | 110 (1.5%) | 233 (3.3%) | 343 (2.4%) |
| Other neurological disorders | 493 (6.9%) | 1015 (14.2%) | 1508 (10.5%) |
| Chronic pulmonary disease | 1804 (25.2%) | 2275 (31.7%) | 4079 (28.5%) |
| Diabetes | 2334 (32.6%) | 2855 (39.8%) | 5189 (36.2%) |
| Hypothyroidism | 1255 (17.5%) | 1279 (17.8%) | 2534 (17.7%) |
| Renal failure | 1733 (24.2%) | 2311 (32.2%) | 4044 (28.2%) |
| Liver disease | 754 (10.5%) | 1038 (14.5%) | 1792 (12.5%) |
| Peptic ulcer disease | 125 (1.7%) | 195 (2.7%) | 320 (2.2%) |
| AIDS/HIV | 61 (0.9%) | 41 (0.6%) | 102 (0.7%) |
| Lymphoma | 177 (2.5%) | 182 (2.5%) | 359 (2.5%) |
| Metastatic cancer | 482 (6.7%) | 496 (6.9%) | 978 (6.8%) |
| Solid tumor without metastasis | 2116 (29.5%) | 2128 (29.7%) | 4244 (29.6%) |
| RA/collagen vascular disease | 505 (7.0%) | 469 (6.5%) | 974 (6.8%) |
| Coagulopathy | 528 (7.4%) | 882 (12.3%) | 1410 (9.8%) |
| Obesity | 1356 (18.9%) | 1504 (21.0%) | 2860 (20.0%) |
| Weight loss | 536 (7.5%) | 893 (12.5%) | 1429 (10.0%) |
| Fluid and electrolyte disorders | 1535 (21.4%) | 2430 (33.9%) | 3965 (27.7%) |
| Blood loss anemia | 112 (1.6%) | 177 (2.5%) | 289 (2.0%) |
| Deficiency anemia | 915 (12.8%) | 1290 (18.0%) | 2205 (15.4%) |
| Alcohol abuse | 232 (3.2%) | 422 (5.9%) | 654 (4.6%) |
| Drug abuse | 271 (3.8%) | 365 (5.1%) | 636 (4.4%) |
| Psychoses | 45 (0.6%) | 156 (2.2%) | 201 (1.4%) |
| Depression | 1523 (21.3%) | 1942 (27.1%) | 3465 (24.2%) |
| Cerebrovascular disease | 780 (10.9%) | 1040 (14.5%) | 1820 (12.7%) |
| Previous TBI | 64 (0.9%) | 116 (1.6%) | 180 (1.3%) |
| Sensory impairment | 368 (5.1%) | 412 (5.7%) | 780 (5.4%) |
| Previous delirium | 13 (0.2%) | 43 (0.6%) | 56 (0.4%) |
| Continuous variables are summarized as median (IQR) and categorical variables as n (%). Comorbidity data was measured using data from 1 year before admission. Abbreviations: AIDS = acquired immunodeficiency syndrome, ECI = Elixhauser comorbidity index; HIV = human immunodeficiency virus, RA = rheumatoid arthritis, TBI = traumatic brain injury. | | | |

| Table S6. Sociodemographic and surgical characteristics of confusion patients, by institution | | | | |
| --- | --- | --- | --- | --- |
| **Variable** | **Institution A (N=3185)** | **Institution B (N=1328)** | **Institution C (N=1737)** | **Total**  **(N=6250)** |
| Age | 67.0 (59.0-74.0) | 67.0 (59.0-73.0) | 71.0 (62.0-79.0) | 68.0 (60.0-76.0) |
| Sex |  |  |  |  |
| Female | 1523 (47.8%) | 584 (44.0%) | 937 (53.9%) | 3044 (48.7%) |
| Male | 1662 (52.2%) | 744 (66.0%) | 800 (46.1%) | 3206 (51.3%) |
| Race |  |  |  |  |
| Asian | 19 (0.6%) | 17 (1.3%) | 2 (0.1%) | 38 (0.6%) |
| Black | 635 (19.9%) | 117 (8.8%) | 71 (4.1%) | 823 (13.2%) |
| Other | 9 (0.3%) | 0 (0%) | 4 (0.2%) | 13 (0.2%) |
| White | 2504 (78.6%) | 1190 (89.6%) | 1653 (95.2%) | 5347 (85.6%) |
| Unknown | 18 (0.6%) | 4 (0.3%) | 7 (0.4%) | 29 (0.5%) |
| Insurance |  |  |  |  |
| Private | 2438 (76.5%) | 1040 (78.3%) | 1497 (86.2%) | 4975 (79.6%) |
| Public | 722 (22.7%) | 285 (21.5%) | 227 (13.1%) | 1234 (19.7%) |
| Uninsured | 25 (0.8%) | 3 (0.2%) | 13 (0.7%) | 41 (0.7%) |
| BMI | 27.7 (23.8-32.9) | 26.6 (22.6-31.2) | 27.8 (23.2-33.7) | 27.5 (23.4-32.8) |
| Smoking Status |  |  |  |  |
| Current | 488 (15.3%) | 165 (12.4%) | 312 (18.0%) | 965 (15.4%) |
| Former | 1420 (44.6%) | 607 (45.7%) | 749 (43.1%) | 2776 (44.4%) |
| Never | 1277 (40.1%) | 556 (41.9%) | 676 (38.9%) | 2509 (40.1%) |
| ASA class |  |  |  |  |
| 1-2 | 250 (7.8%) | 36 (2.7%) | 195 (11.2%) | 481 (7.7%) |
| 3-4 | 2535 (79.6%) | 1121 (84.4%) | 1318 (75.9%) | 4974 (79.6%) |
| 5 or E | 400 (12.6%) | 171 (12.9%) | 224 (12.9%) | 795 (12.7%) |
| Surgical Specialty |  |  |  |  |
| CT | 589 (18.5%) | 125 (9.4%) | 140 (8.1%) | 854 (13.7%) |
| ENT | 63 (2.0%) | 49 (3.7%) | 51 (2.9%) | 163 (2.6%) |
| General | 376 (11.8%) | 693 (52.2%) | 405 (23.3%) | 1474 (23.6%) |
| Multiple | 234 (7.3%) | 119 (9.0%) | 50 (2.9%) | 403 (6.4%) |
| Neurology | 783 (24.6%) | 5 (0.4%) | 233 (13.4%) | 1021 (16.3%) |
| Orthopedics | 487 (15.3%) | 52 (3.9%) | 513 (29.5%) | 1052 (16.8%) |
| Other | 18 (0.6%) | 36 (2.7%) | 24 (1.4%) | 78 (1.2%) |
| Plastics | 82 (2.6%) | 16 (1.2%) | 87 (5.0%) | 185 (3.0%) |
| Urogyn | 172 (5.4%) | 227 (17.1%) | 119 (6.9%) | 518 (8.3%) |
| Vascular | 518 (13.9%) | 375 (10.0%) | 5 (0.3%) | 2 (0.1%) |
| Confusion patients had nurse-documented confusion but no documented delirium (CAM or ICD code). Continuous variables are summarized as median (IQR) and categorical variables as n (%). Abbreviations: BMI, body mass index; ASA, American Society of Anesthesiologists; CT, cardiothoracic, ENT, ears, nose, and throat; uro/gyn = urology gynecology. | | | | |

| Table S7. Clinical characteristics of confusion patients, by institution | | | | |
| --- | --- | --- | --- | --- |
| **Variables** | **Institution A (N=3185)** | **Institution B (N=1328)** | **Institution C (N=1737)** | **Total**  **(N=6250)** |
| ECI Score | 5 (0-14) | 11 (4-21) | 5 (0-14) | 6 (0-16) |
| Number of ICD codes | 20 (11-33) | 22 (11-36.3) | 18 (9-32) | 20 (10-34) |
| Comorbidities |  |  |  |  |
| Congestive heart failure | 645 (20.3%) | 151 (11.4%) | 385 (22.2%) | 1181 (18.9%) |
| Cardiac arrhythmias | 831 (26.1%) | 318 (23.9%) | 483 (27.8%) | 1632 (26.1%) |
| Valvular disease | 581 (18.2%) | 107 (8.1%) | 176 (10.1%) | 864 (13.8%) |
| Pulmonary circulation disorders | 267 (8.4%) | 85 (6.4%) | 112 (6.4%) | 464 (7.4%) |
| Peripheral vascular disorders | 852 (26.8%) | 179 (13.5%) | 395 (22.7%) | 1426 (22.8%) |
| Hypertension | 2193 (68.9%) | 830 (62.5%) | 1152 (66.3%) | 4175 (66.8%) |
| Paralysis | 85 (2.7%) | 17 (1.3%) | 58 (3.3%) | 160 (2.6%) |
| Other neurological disorders | 353 (11.1%) | 141 (10.6%) | 178 (10.2%) | 672 (10.8%) |
| Chronic pulmonary disease | 851 (26.7%) | 345 (26.0%) | 541 (31.1%) | 1737 (27.8%) |
| Diabetes | 1127 (35.4%) | 425 (32.0%) | 642 (37.0%) | 2194 (35.1%) |
| Hypothyroidism | 551 (17.3%) | 228 (17.2%) | 316 (18.2%) | 1095 (17.5%) |
| Renal failure | 767 (24.1%) | 385 (29.0%) | 362 (20.8%) | 1514 (24.2%) |
| Liver disease | 243 (7.6%) | 352 (26.5%) | 106 (6.1%) | 701 (11.2%) |
| Peptic ulcer disease excluding bleeding | 47 (1.5%) | 38 (2.9%) | 41 (2.4%) | 126 (2.0%) |
| AIDS/HIV | 36 (1.1%) | 9 (0.7%) | 2 (0.1%) | 47 (0.8%) |
| Lymphoma | 61 (1.9%) | 50 (3.8%) | 31 (1.8%) | 142 (2.3%) |
| Metastatic cancer | 136 (4.3%) | 215 (16.2%) | 71 (4.1%) | 422 (6.8%) |
| Non-metastatic cancer | 638 (20.0%) | 673 (50.7%) | 341 (19.6%) | 1652 (26.4%) |
| RA/collagen vascular disease | 170 (5.3%) | 59 (4.4%) | 132 (7.6%) | 361 (5.8%) |
| Coagulopathy | 221 (6.9%) | 169 (12.7%) | 152 (8.8%) | 542 (8.7%) |
| Obesity | 555 (17.4%) | 209 (15.7%) | 386 (22.2%) | 1150 (18.4%) |
| Weight loss | 237 (7.4%) | 198 (14.9%) | 146 (8.4%) | 581 (9.3%) |
| Fluid/electrolyte disorders | 732 (23.0%) | 416 (31.3%) | 460 (26.5%) | 1608 (25.7%) |
| Blood loss anemia | 48 (1.5%) | 37 (2.8%) | 25 (1.4%) | 110 (1.8%) |
| Deficiency anemia | 450 (14.1%) | 240 (18.1%) | 289 (16.6%) | 979 (15.7%) |
| Alcohol abuse | 128 (4.0%) | 86 (6.5%) | 51 (2.9%) | 265 (4.2%) |
| Drug abuse | 159 (5.0%) | 52 (3.9%) | 71 (4.1%) | 282 (4.5%) |
| Psychoses | 50 (1.6%) | 30 (2.3%) | 44 (2.5%) | 124 (2.0%) |
| Depression | 710 (22.3%) | 300 (22.6%) | 411 (23.7%) | 1421 (22.7%) |
| Cerebrovascular disease | 488 (15.3%) | 79 (5.9%) | 205 (11.8%) | 772 (12.4%) |
| Previous TBI | 62 (1.9%) | 13 (1.0%) | 40 (2.3%) | 115 (1.8%) |
| Sensory impairment | 167 (5.2%) | 68 (5.1%) | 86 (5.0%) | 321 (5.1%) |
| Previous delirium | 307 (9.6%) | 152 (11.4%) | 189 (10.9%) | 648 (10.4%) |
| Continuous variables are summarized as median (IQR) and categorical variables as n (%). Comorbidity data measured using data from 1 year before admission. Confusion patients had nurse-documented confusion but no documented delirium (CAM or ICD code). Abbreviations: AIDS = acquired immunodeficiency syndrome, ECI = Elixhauser comorbidity index; HIV = human immunodeficiency virus, RA = rheumatoid arthritis, TBI = traumatic brain injury. | | | | |

| Table S8. Top 5 most influential variables used by XGB models, 6-month surveillance period | | | | |
| --- | --- | --- | --- | --- |
|  |  | **Holdout Dataset** | | |
| **Model** | **Rank** | **Institution A** | **Institution B** | **Institution C** |
| XGBa | 1 | ASA class | ASA class | ASA class |
|  | 2 | ICD group: Z00-Z13 | ICD group: Z00-Z13 | ICD group: Z00-Z13 |
|  | 3 | Multispecialty surgery | Multispecialty surgery | Service: hospitalist |
|  | 4 | Service: hospitalist | Service: hospitalist | Emergency surgery |
|  | 5 | Emergency surgery | Emergency surgery | Multispecialty surgery |
| XGBb | 1 | ASA class | ASA class | ASA class |
|  | 2 | Multispecialty surgery | Multispecialty surgery | Uro/gyn surgery |
|  | 3 | Uro/gyn surgery | Uro/gyn surgery | Multispecialty surgery |
|  | 4 | ATC A06 | ATC A06 | Number of comorbidities |
|  | 5 | Number of comorbidities | ICD group: Z69-Z76 | ATC A06 |
| XGBc | 1 | ASA class | ASA class | ASA class |
|  | 2 | Service: hospitalist | Service: hospitalist | Service: orthopedics |
|  | 3 | ATC B01 | Service: orthopedics | Service: hospitalist |
|  | 4 | Service: orthopedics | ATC B01 | ATC B01 |
|  | 5 | Emergency surgery | Emergency surgery | Emergency surgery |
| Feature importance measured using SHAP values. XGB_A_ , XGB_b_ , and XGB_c_ were trained on data from institutions A, B, and C, respectively. ASA, American Society of Anesthesiologists; ICD group Z00-Z13, persons encountering health services for examinations. ICD group Z77-Z99, persons with potential health hazards related to family and personal history and certain conditions influencing health status; service: hospitalist, admitted to hospitalist service; service: orthopedics, admitted to orthopedics service; Uro/gyn, urology/gynecology. ATC A06 = anatomic therapeutic chemicals group A06 constipation medications. ATC B01 = anatomic therapeutic chemicals group B01 antithrombotic medications. | | | | |

| Table S9. Top 5 most influential variables used by XGB models, 3-month surveillance period | | | | |
| --- | --- | --- | --- | --- |
|  |  | **Holdout Dataset** | | |
| **Model** | **Rank** | **Institution A** | **Institution B** | **Institution C** |
| XGBa | 1 | ASA class | ASA class | ASA class |
|  | 2 | Multispecialty surgery | Multispecialty surgery | Service: hospitalist |
|  | 3 | ICD group: Z00-Z13 | ICD group: Z00-Z13 | ICD group: Z00-Z13 |
|  | 4 | Service: hospitalist | Service: hospitalist | Emergency surgery |
|  | 5 | Emergency surgery | Emergency surgery | Multispecialty surgery |
| XGBb | 1 | ASA class | ASA class | ASA class |
|  | 2 | Multispecialty surgery | Multispecialty surgery | Multispecialty surgery |
|  | 3 | Number of comorbidities | Uro/gyn surgery | Number of comorbidities |
|  | 4 | Uro/gyn surgery | ATC A06 | Uro/gyn surgery |
|  | 5 | ATC A06 | Number of comorbidities | ATC A06 |
| XGBc | 1 | ASA class | Service: hospitalist | Service: hospitalist |
|  | 2 | Service: hospitalist | ASA class | Service: orthopedics |
|  | 3 | Service: orthopedics | Service: orthopedics | ASA class |
|  | 4 | Number of medications | Number of medications | Number of medications |
|  | 5 | Emergency surgery | Emergency surgery | Emergency surgery |
| Feature importance measured using SHAP values. XGB_A_ , XGB_b_ , and XGB_c_ were trained on data from institutions A, B, and C, respectively. ASA, American Society of Anesthesiologists; BMI, body mass index; ICD group Z00-Z13, persons encountering health services for examinations. ICD group Z77-Z99, persons with potential health hazards related to family and personal history and certain conditions influencing health status; service: hospitalist, admitted to hospitalist service; service: orthopedics, admitted to orthopedics service; ATC, anatomic therapeutic chemicals classification; Uro/gyn, urology/gynecology; ATC A06 = anatomic therapeutic chemicals group A06 constipation medications. | | | | |

| Table S10. XGB model predictions for confusion encounters | | | | |
| --- | --- | --- | --- | --- |
| **Surveillance Period** | **Model** | **Data** | **Predicted Case** | **Predicted Control** |
| 1 year | XGBa | Inst. A | 1447/3185 (45.4%) | 1738/3185 (54.6%) |
|  |  | Inst. B | 499/1328 (37.6%) | 829/1328 (62.4%) |
|  |  | Inst. C | 923/1737 (53.1%) | 814/1737 (46.9%) |
|  | XGBb |  |  |  |
|  |  | Inst. A | 1884/3185 (59.2%) | 1301/3185 (40.8%) |
|  |  | Inst. B | 676/3128 (50.9%) | 652/1328 (49.1%) |
|  |  | Inst. C | 875/1737 (50.4%) | 682/1737 (49.6%) |
|  | XGBc |  |  |  |
|  |  | Inst. A | 1752/3185 (55.0%) | 1433/3185 (45.0%) |
|  |  | Inst. B | 632/1328 (47.6%) | 696/1328 (52.4%) |
|  |  | Inst. C | 930/1737 (53.5%) | 807/1737 (46.5%) |
| 6 months | XGBa | Inst. A | 1394/3185 (43.8%) | 1791/3185 (56.2%) |
|  |  | Inst. B | 504/1328 (38% | 824/1328 (62.0%) |
|  |  | Inst. C | 903/1737 (52.0%) | 834/1737 (48.0%) |
|  |  |  |  |  |
|  | XGBb | Inst. A | 1869/3185 (58.7%) | 1316/3185 (41.3%) |
|  |  | Inst. B | 698/1328 (52.6%) | 630/1328 (47.4%) |
|  |  | Inst. C | 838/1737 (48.2%) | 899/1737 (51.8%) |
|  |  |  |  |  |
|  | XGBc | Inst. A | 1897/3185 (59.6%) | 1288/3185 (40.4%) |
|  |  | Inst. B | 598/1328 (45.0%) | 730/1328 (55.0%) |
|  |  | Inst. C | 954/1737 (54.9%) | 783/1737 (45.1%) |
| 3 months | XGBa | Inst. A | 1451/3185 (45.6%) | 1734/3185 (54.4%) |
|  |  | Inst. B | 511/1328 (38.4%) | 817 (61.5%) |
|  |  | Inst. C | 916/1737 (52.7%) | 821/1737 (47.3%) |
|  |  |  |  |  |
|  | XGBb | Inst. A | 1809/3185 (56.8%) | 1376/3185 (43.2%) |
|  |  | Inst. B | 711/1328 (53.5%) | 617/1328 (46.5%) |
|  |  | Inst. C | 858/1737 (49.4%) | 879/1737 (50.6%) |
|  |  |  |  |  |
|  | XGBc | Inst. A | 1819/3185 (57.1%) | 1366/3185 (42.9%) |
|  |  | Inst. B | 566/1328 (42.6%) | 762/1328 (57.4%) |
|  |  | Inst. C | 1054/1737 (60.7%) | 683/1737 (39.3%) |
| Confusion encounters are encounters with nurse-documented confusion but no documented delirium. Inst. = institution. | | | | |
